# RTPDB: a database providing associations between genetic variation or expression and cancer prognosis with radiotherapy-based treatment

**DOI:** 10.1093/database/bay118

**Published:** 2018-10-30

**Authors:** Cheng-Dong Zhang, Yuan Yang, Huan-Huan Chen, Ting Zhang, Qiang Wang, Yuan Liang, Liang Zhang, Yan Zhou

**Affiliations:** 1 State Key Laboratory of Genetic Engineering, School of Life Sciences, Fudan University, Shanghai, China; 2 School of Preclinical Medicine, Guangxi Medical University, Nanning, Guangxi, China; 3 School of Mathematics and Physics, Anhui University of Technology, Maanshan, Anhui, China; 4 Shanghai-MOST Key Laboratory of Health and Disease Genomics, Chinese National Human Genome Center at Shanghai, Shanghai, China

## Abstract

In recent years, lots of studies have reported the relationship between genetic variation or expression and cancer prognosis with radiotherapy-based treatment. However, due to limitation in available journals or literature database, inconsistent nomenclature system of genetic variation and cancer and time-consuming investigation on literature searching and reading, considerable researches could hardly get found and cited. In this study, we constructed the Radiotherapy Prognosis Database (RTPDB), which contains a comprehensive resource about genes and related cancer prognosis. It included 775 studies, which consist of 275 Single Nucleotide Polymorphism (SNP) studies with 59 765 patients, 261 genes, 708 SNPs, 16 tumors and 16 treatment types, and 500 expression studies with 55 751 patients, 264 genes, 27 tumors and 15 treatment types. The names of genes and their variants were converted and displayed in the form of the official symbol. The detailed information of the tumor, treatment and prognosis were classified. We hope RTPDB will be a useful resource with great potential for researches on genes, variants and cancer prognosis.

## Introduction

Radiotherapy is a common treatment for cancer today, alone or in combination with other treatments. According to the American Society of Radiation Oncology, >60% of cancer patients will receive radiotherapy—radiotherapy using high-energy radiation to shrink tumors and kill cancer cells—contributing to 40% of curative treatment for cancer (1). When the DNA of a cancer cell is damaged by radiation, it will stop dividing or it will die, and then it will be eliminated by the immune system. As surgery will inevitably remove normal tissues, chemotherapy has its drawbacks in unavoidably killing normal cells. Radiotherapy not only kills cancer cells but also affects normal cells around the cancer cells, and further leads to side effects. Based on the time of occurrence, it can be divided into acute and late side effects. Various cancer patients have different sensitivity to radiotherapy. In general, high sensitivity to radiotherapy and mild side effects are important for long-term survival (2).

Analogous to pharmacogenomics, the term radiogenomics is used to explain the differences in radiotherapy response between individuals. In 2009, a Radiogenomics Consortium was established to facilitate and promote multi-center collaboration of researchers linking genetic variation with response to radiotherapy (3). It might lead to improved decision making, and as a result, improved patient outcomes (4). During the past years, thousands of researches have studied the relationship between genetic variation or expression and patient prognosis who received radiotherapy-based treatment. Most of them focus on genes which take part in cell growth, differentiation, proliferation, and apoptosis, such as XRCC1 (5) and TP53 (6). Unlike pharmacogenomics, radiogenomics studies now lack a database similar to PharmGKB, which is responsible for the aggregation, curation, integration and dissemination of knowledge regarding the impact of human genetic variation on drug response (7). Due to the relatively small number of radiogenomic studies compared to the pharmacogenomics and the dispersion of the literature, the advances in radiogenomics have been hindered. Most of the researches could hardly get found and cited. Therefore, a high-quality resource platform with unlimited use, standard nomenclature and convenient searching process is believed to be of great value in the understanding of gene variants or expression and cancer prognosis under radiotherapy.

In this paper, we describe the Radiotherapy Prognosis Database (RTPDB), a comprehensive online database established to collect the associations between genetic variants or expression and cancer prognosis of patients who received radiotherapy-based treatment and were documented in biomedical literature. It is the first database for genes and related cancer radiotherapy prognosis. The database offers exciting opportunities for scientists and clinicians to better explore the overview of the relationship between genes and related cancer radiotherapy prognosis. In addition, it will be helpful for researchers to understand the mechanism of cancer prognosis with radiation treatment. The RTPDB can be publicly accessed from http://www.rtpdb.com/.

## Materials and methods

### Software design and implementation

In RTPDB database, all data sets were organized in our web server using the client–server model based on Python, Django, JavaScript and PostgreSQL. The database is available at http://www.rtpdb.com/. RTPDB contains pages for searching, browsing and downloading.

### Literature searching and inclusion

In addition to the common database such as China Knowledge Infrastructure, PubMed, Web of Science, EMBASE and Google Scholar, we also conducted a detailed manual review for the references of the studies included in the database by two different authors (C.D.Z. and Y.Y.). Articles published before August 2018 were searched with a combination of mesh term and keywords as follows: Single Nucleotide Polymorphisms, Gene Expression Neoplasms, Radiotherapy and Prognosis. No restriction on publication language, year or geographic region was imposed. All eligible studies were retrieved with the PDF file. The literature was included according to the following criteria: (1) studying the relationship between gene variants or expression and cancer prognosis, (2) patients received radiotherapy with or without other treatments and (3) using one of the statistics that Odds Ratio (OR), Hazard Radio (HR) and Risk ratio (RR) and their 95% Confidence Interval (CI) to evaluate the relationship.

### Alias conversion for genes and variants

We converted all the gene symbols into the official symbols with NCBI Gene database (8) and used Gene ID to get the official full name, gene type, alias, summary and gene pathways provided by MyGene.info (9). For variants, the HGVS (Humane Genome Variation Society) name or else were converted into dbSNP RS ID with Google search, SNPedia (10) and NCBI dbSNP database (8). The allele frequency of each SNP in African, Ad Mixed American, East Asian, European and South Asian were collected from 1000 Genomes (11).

### Classification for treatment, tumor and prognosis

For treatment, we first confirm the type of treatment the patients received, for example, radiotherapy, chemotherapy, surgery and hormone therapy. Then, we need to confirm whether all patients received the above treatments. Finally, the treatment could be classified into `Radiotherapy +/± Chemotherapy +/± Surgery +/± Hormone therapy’, which means all patients received radiotherapy and all (±)/partial (±) patients received other treatments.

A few tumors (or subtype) do not have corresponding medical subject headings. Therefore, all tumors were classified according to the location of tumor primary lesion. For example, esophageal squamous cell carcinoma and esophageal adenocarcinoma were classified into esophageal cancer.

Prognosis mainly includes three types: treatment response, survival and side effect. Treatment response indicates the tumor regression after radiotherapy-based treatment. Survival stands for the time from the end of the treatment to death, such as Overall Survival, Recurrence-Free Survival, PFS Progression-Free Survival, Metastasis-Free Survival and Cancer-Specific Survival.

### Data extraction

Aside from the above information, we also included the following data: patients’ clinical information including total patient number, age (median, mean and range), sex ratio, ethnicity, tumor stage and patient number in each stage, study’s basic information including publication data (year, type and journal), language, abstract and the relationship between genetic variants or expression and prognosis. In expression studies, we also collected the detection method and cut-off value of gene expression. All data were manually curated and collected. Some studies do not provide the complete information, so you may get `Not Provided’ in some fields.

### Calculating OR

Partial studies use Fisher’s exact test to evaluate the association between gene variants or expression and cancer prognosis. Compared with Fisher’s exact test, OR could quantify the association with 95% CI. We use data in 4-fold table (Table 1) and calculate OR and 95% CI by STATA 14.1 (StataCorp. 2015. Stata Statistical Software: Release 14. College Station, TX: StataCorp LP.).

**Table 1:** 4-fold table

	Event	Non-event
Exposure	a	b
Non-exposure	c	d

Note: $\mathrm{OR}=\frac{a/b}{c/d}=\frac{ad}{bc};\mathrm{HR}=\frac{a/\left(a+b\right)}{c/\left(c+d\right)}=\frac{a\left(c+d\right)}{c\left(a+b\right)}\ \left( in\ a\  period\ of\ time\right);\mathrm{RR}=\frac{a/\left(a+b\right)}{c/\left(c+d\right)}=\frac{a\left(c+d\right)}{c\left(a+b\right)}$

### Gene Ontology and Pathway Enrichment Analysis

After collecting the studies matching the criteria of our study, we extracted all the significant genes (SNPs were annotated by their genes) for further analysis. Gene ontology and pathway enrichment analysis were performed on DAVID 6.8 (https://david.ncifcrf.gov/). The enrichment *P* values of both GO (Gene Ontology) and pathway enrichment analyses were set as significant when *P* < 0.05. GO analysis and pathway analysis result table can be accessed in the Download page of the website.

## Results and discussion

### The RTPDB web interface

**Figure 1:**
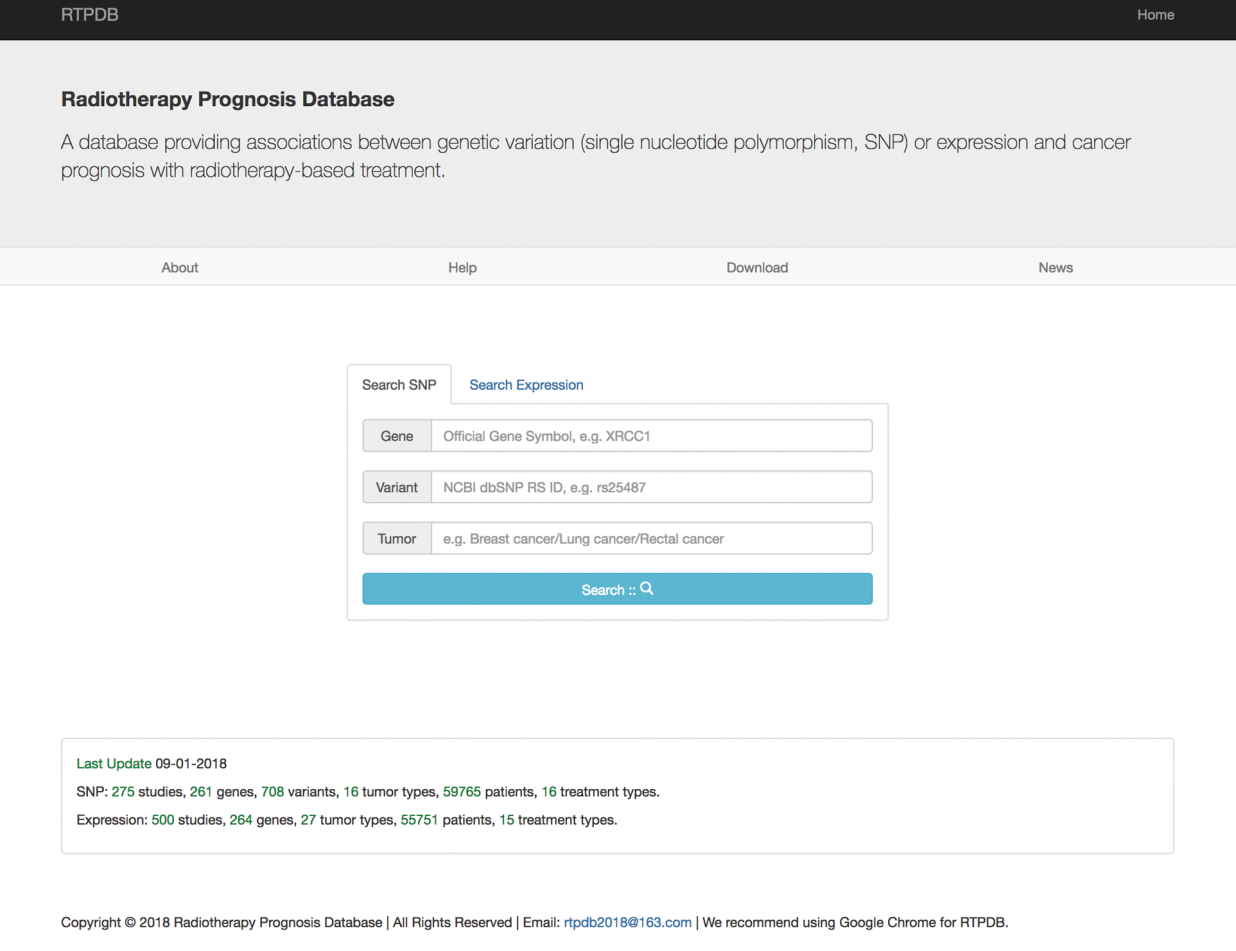
The RTPDB use interface showing the homepage.

The data in RTPDB can be easily accessed from http://www.rtpdb.com/. First, users need to choose `Search SNP’ or `Search Expression’ at homepage (Figure 1). Second, enter their interested gene, variant and tumor and click `Search’. Then click the literature title and the corresponding result will be shown on the result page. In the result page, users can acquire study information, clinical information of patients, treatment types, allele frequency of SNP and the relationship between genetic variants or expression and prognosis (OR/HR/RR with 95% CI). Besides, when you search `Expression’, the RTPDB provides the detection method and cut-off value of gene expression. The database also provides hyperlinks to original references for each included study. The website is compatible with Google Chrome, and we highly recommend using Google Chrome for RTPDB.

### Data included in the database

The literature search yielded >18 000 publications. To meet the need of RTPDB construction, we selected literatures that provide the association between gene variants or gene expression and cancer prognosis of patients who received radiotherapy-based treatment. More importantly, the associations were evaluated by OR, HR and RR with 95% CI. After filtering, the studies unable to meet the inclusion criteria were excluded based on title, abstract or method and result.

**Figure 2:**
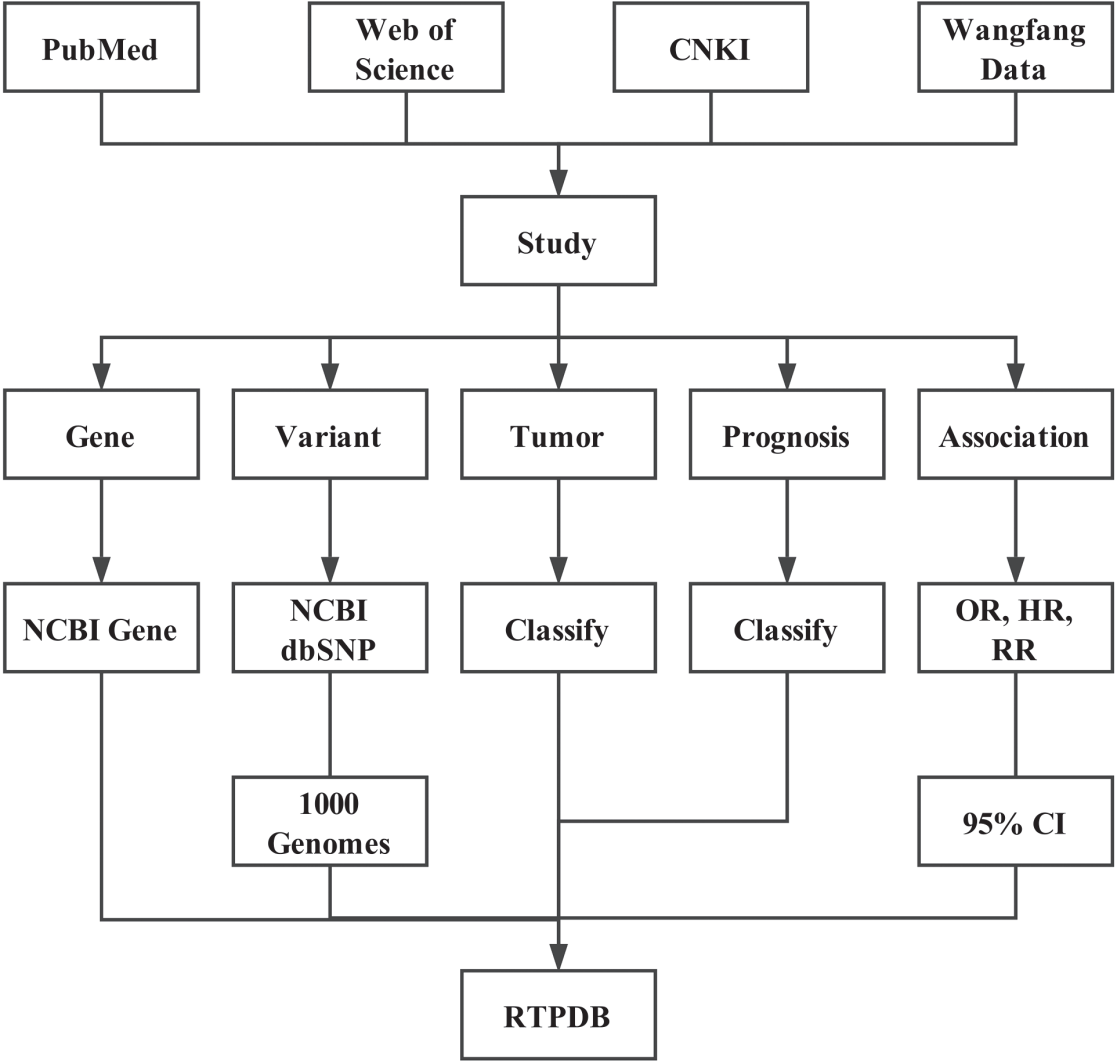
Flowchart of the RTPDB.

**Figure 3:**
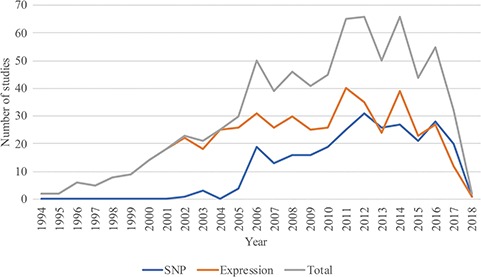
The distribution of studies included in the database from 1994 to 2018.

**Figure 4:**
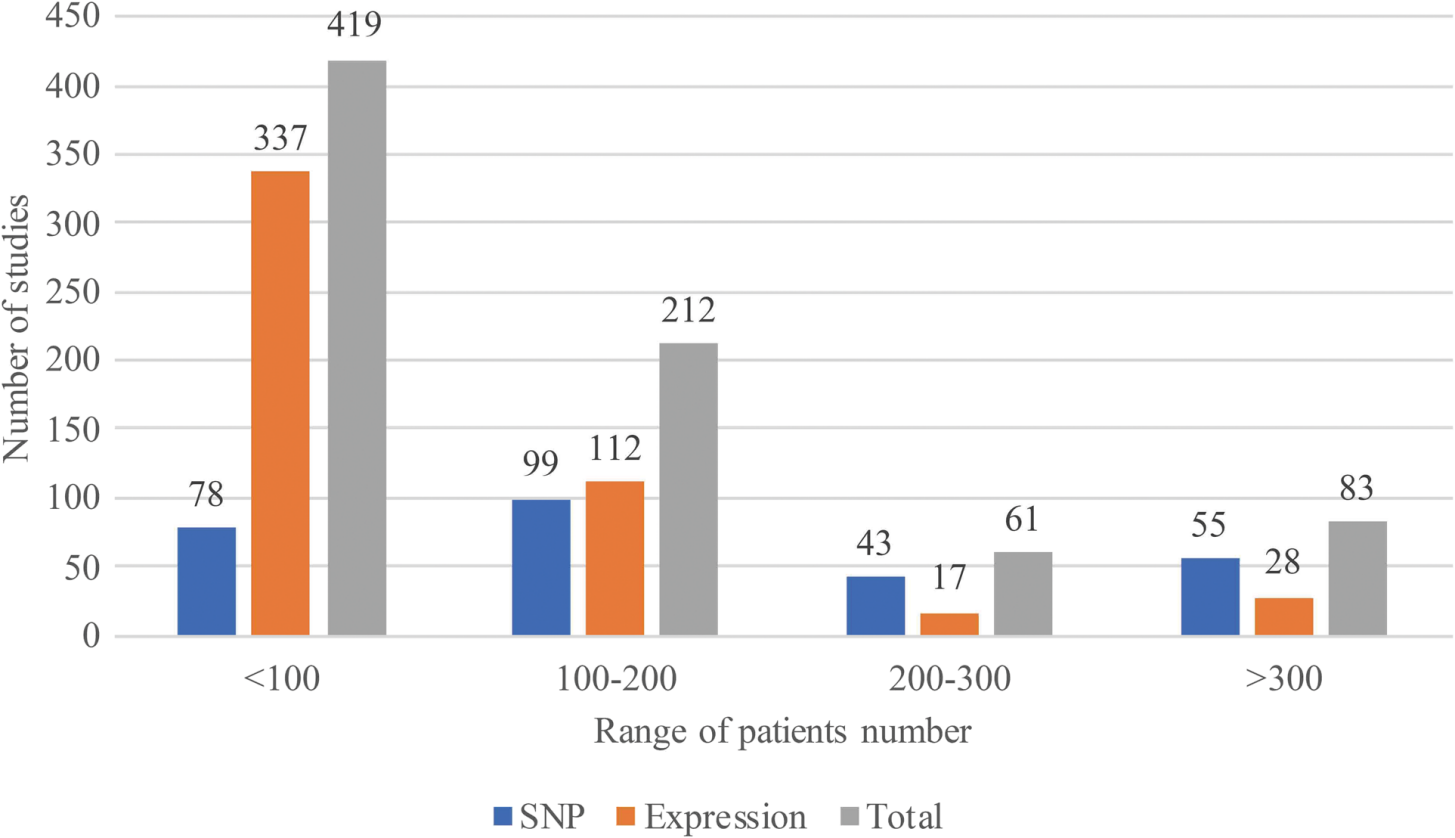
The range of patients’ number in included studies.

In August 2018, RTPDB included 775 studies, which consist of 500 expression studies and 275 SNP studies (Figure 2). These included studies dated from 1994 to 2018 and the number of studies published per year showed an increasing trend from 2002 to 2012 (Figure 3). Most articles were written in English and published in journals (Supplementary Table 1). Database included 115 516 patients which consist of 59 765 patients in variant studies and 55 751 patients in expression studies. The number of patients in most studies is <200 (Figure 4). A study with 150 patients will only have a power of <30% to detect the association (12). It is unlikely to get a more comprehensive result with a small sample size, so further researches should include more patients.

**Figure 5:**
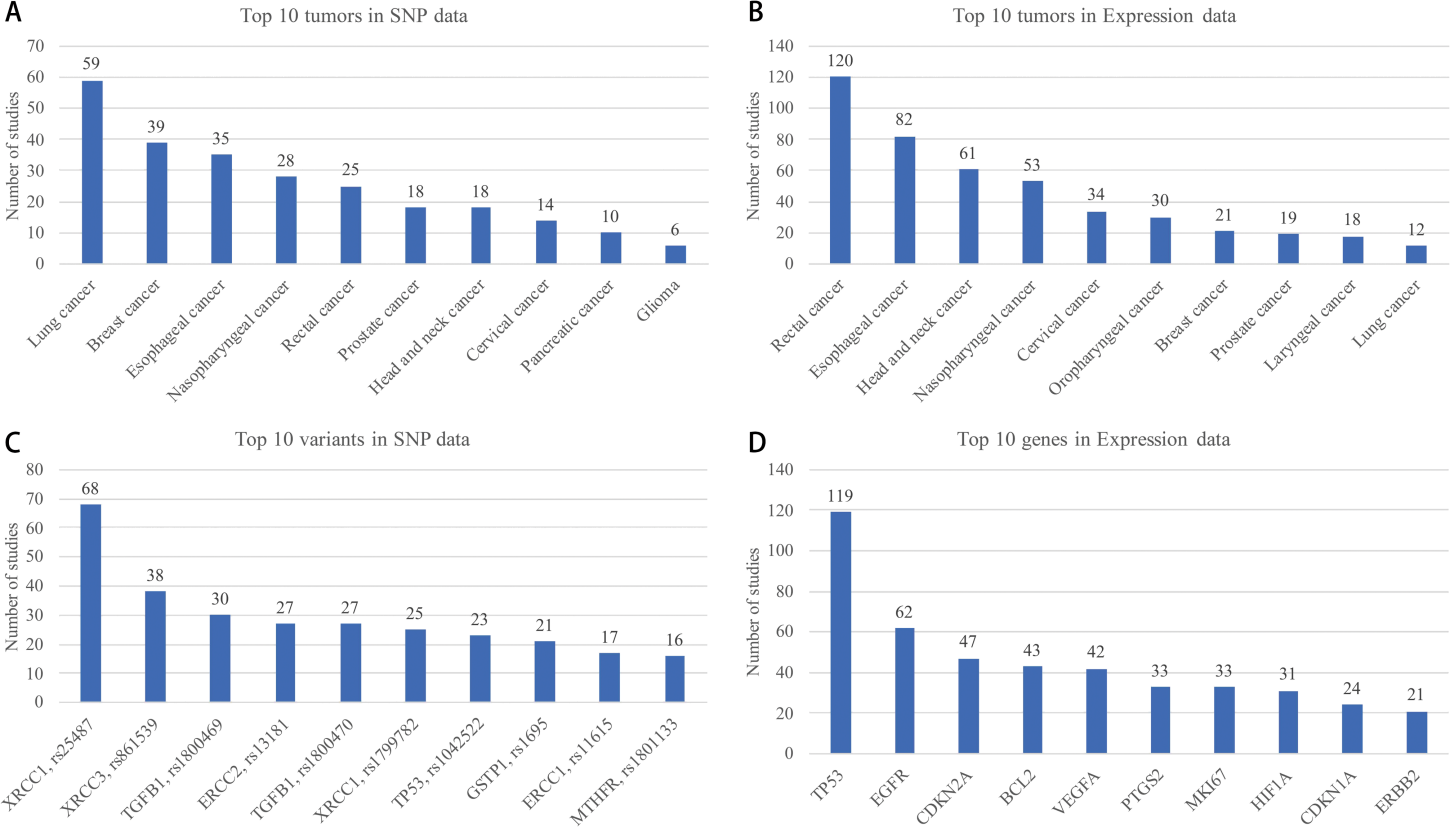
Top 10 tumor, variant and gene in SNP and expression data.

Top 10 tumors in SNP and Expression are shown in Figure 5A and B, respectively. Breast cancer, lung cancer, esophageal cancer and nasopharyngeal cancer are the most studied cancers, which is basically consistent with the morbidity and mortality in many reports of cancer statistics. A total of 275 variant studies included 261 genes and 708 SNPs, and 500 expression studies included 264 genes. Interestingly, only one of the 700 SNPs is located in the intergenic region. Most studies have paid attention to genes that take part in the important pathways such as cell cycle. However, the intergenic region of a genome may have influences on the function of genes. Top 10 SNPs and genes which have been studied are shown in Figure 5C and D, respectively.

**Figure 6:**
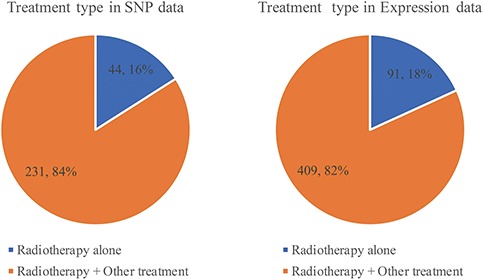
The distribution of treatment type in SNP and expression data.

Less than one-fifth of studies use radiotherapy alone. Most of them choose radiotherapy combination with chemotherapy, surgery and hormone therapy (Figure 6, Supplementary Tables 2 and 3). Whether before or after surgery, radiotherapy will shrink the tumor so that the risk of recurrence will be reduced. Each relationship contains four major items, which are tumor, gene with variant or expression, prognosis with endpoint and OR/HR/RR with 95% CI. RTPDB consists of 2608 relationships between SNPs and prognosis and 1874 relationships between gene expression and prognosis.

The significant genes were combined together and input to DAVID web server. Top 15 biological process (such as DNA repair, response to X-ray), cellular component (such as nucleoplasm, replication fork) and molecular function (such as damaged DNA binding, double-stranded DNA binding) were plotted in Supplementary Figure 1. For pathway analysis, we excluded pathways associated with specific tumors and left those pathways that are more instructive for biological processes, such as VEGF signaling pathway, T cell receptor signaling pathway, etc. The detailed figure was shown in Supplementary Figure 2.

## Conclusion

Thousands of studies have reported the relationship between SNPs or gene expression and cancer prognosis of patients who received radiotherapy-based treatment. In this article, we developed the RTPDB database to collect and curate the associations. The number of entries in RTPDB is not very large. That is because of the follow up of cancer patients after treatment is time-consuming. However, many researchers began to realize the importance of gene variants and expression in cancer prognosis. The prognosis-related gene and variant may become the biomarker for personalized treatment. With the development of studies, more cancer prognosis-related genes and variants are expected to be published and included into RTPDB. The purpose of RTPDB is to provide comprehensive resource about the association between gene variants or expression and cancer prognosis.

Next step, we plan to update RTPDB every 2 months with new published studies, and other types of variants will be included if the number of studies is enough. Meanwhile, the open access data related to radiotherapy in The Cancer Genome Atlas, Gene Expression Omnibus and Sequence Read Archive will be integrated into the RTPDB database. We believe that RTPDB would be useful for the studies focusing on the associations among gene variants or expression and cancer prognosis and will offer more help when more data were included in the future.

### Availability

RTPDB database is freely available at http://www.rtpdb.com/.

## Supplementary Material

Supplementary Data

## Notes

**Database URL:** http://www.rtpdb.com/

## References

[1] Barnett G.C., West C.M.L., Dunning A.M. et al. (2009) Normal tissue reactions to radiotherapy: towards tailoring treatment dose by genotype. Nat. Rev. Cancer, 9, 134–142. 19148183 10.1038/nrc2587 PMC2670578

[2] Wang M., Delasalle K., Feng L. et al. (2010) CR represents an early index of potential long survival in multiple myeloma. Bone Marrow Transplant., 45, 498–504. 19633690 10.1038/bmt.2009.176 PMC5777472

[3] West C., Rosenstein B.S., Alsner J. et al. (2010) Establishment of a Radiogenomics Consortium. Radiother. Oncol., 76, 1295–1296. 10.1016/j.ijrobp.2009.12.017 20338472

[4] Mazurowski M.A. (2015) Radiogenomics: what it is and why it is important. J. Am. Coll. Radiol., 12, 862. 26250979 10.1016/j.jacr.2015.04.019

[5] Yin M., Liao Z., Liu Z. et al. (2011) Functional polymorphisms of base excision repair genes XRCC1 and APEX1 predict risk of radiation pneumonitis in patients with non-small cell lung cancer treated with definitive radiation therapy. Int. J. Radiat. Oncol. Biol. Phys., e67, 81. 10.1016/j.ijrobp.2010.11.079 PMC3136565 21420246

[6] Lopezcrapez E., Bibeau F., Thézenas S. et al. (2005) p53 status and response to radiotherapy in rectal cancer: a prospective multilevel analysis. Br. J. Cancer, 92, 2114–2121. 15956964 10.1038/sj.bjc.6602622 PMC2361816

[7] Thorn C.F., Klein T.E. and Altman R.B. (2013) PharmGKB: the pharmacogenomics knowledge base. Methods Mol. Biol., 1015, 311. 23824865 10.1007/978-1-62703-435-7_20 PMC4084821

[8] Sayers E.W., Barrett T., Benson D.A. et al. (2010) Database resources of the national center for biotechnology information. IEEE Haptics Symp., 199–205. 10.1093/nar/gkp967 PMC2808881 19910364

[9] Xin J., Mark A., Afrasiabi C. et al. (2016) High-performance web services for querying gene and variant annotation. Genome Biol., 17, 1–7. 27154141 10.1186/s13059-016-0953-9 PMC4858870

[10] Michael C. and Greg L. (2012) SNPedia: a wiki supporting personal genome annotation, interpretation and analysis. Nucleic Acids Res., 40, 1308–1312. 10.1093/nar/gkr798 PMC3245045 22140107

[11] Consortium T.G.P. (2015) A global reference for human genetic variation. Nature, 526, 68. 26432245 10.1038/nature15393 PMC4750478

[12] Andreassen C.N. and Alsner J. (2009) Genetic variants and normal tissue toxicity after radiotherapy: a systematic review. Radiother. Oncol., 92, 299–309. 19683821 10.1016/j.radonc.2009.06.015

