# Supplementary material for: RTPDB: a database providing associations between genetic variation or expression and cancer prognosis with radiotherapy-based treatment
Source: Database (Oxford). 2018 Oct 30;2018:bay118. doi: 10.1093/database/bay118 (PMC6206893; doi:10.1093/database/bay118)
Supplement: Supplementary Data [file bay118_supp.zip › Supplementary Table.docx]

Supplementary Table 1. The language and publication type of included studies

|  |  | SNP (%) | Expression (%) | Total (%) |
| --- | --- | --- | --- | --- |
| Language | Chinese | 46 (16.72%) | 53 (10.60%) | 99 (12.77%) |
|  | English | 229 (83.28%) | 446 (89.20%) | 665 (87.10%) |
|  | Korean | 0 (0.00%) | 1 (0.20%) | 1 (0.13%) |
| Publication type | Journal Article | 265 (96.37%) | 479 (95.80%) | 744 (96.00%) |
|  | Doctoral Dissertation | 5 (1.82%) | 13 (2.60%) | 18 (2.32%) |
|  | Masters' Thesis | 4 (1.45%) | 8 (1.60%) | 12 (1.55%) |
|  | Proceedings | 1 (0.36%) | 0 (0.00%) | 1 (0.13%) |
| Total | | 275 (35.48%) | 494 (64.52%) | 775 (100%) |

Supplementary Table 2. The treatment types in SNP data

| Treatment type | Meaning | No. of Research (%) |
| --- | --- | --- |
| RT ± CT | All received RT, and partial received CT. | 62 (22.55%) |
| RT + CT | All received RT and CT. | 46 (16.73%) |
| RT | All received RT. | 44 (16.00%) |
| RT + CT + S | All received RT, CT and S. | 36 (13.09%) |
| RT ± CT ± S | All received RT, and partial received CT and S. | 19 (6.91%) |
| RT + S | All received RT and S. | 18 (6.55%) |
| RT + CT ± S | All received RT and CT, and partial received S. | 16 (5.82%) |
| RT ± HT | All received RT, and partial received HT. | 8 (2.91%) |
| RT ± CT + S | All received RT and S, and partial received CT. | 8 (2.91%) |
| RT ± CT ± HT + S | All received RT and S, and partial received CT and HT. | 6 (2.18%) |
| RT ± HT ± S | All received RT, and partial received HT and S. | 5 (1.85%) |
| RT ± HT + S | All received RT and S, and partial received HT. | 2 (0.73%) |
| RT ± CT ± HT | All received RT, and partial received CT and HT. | 2 (0.73%) |
| RT + CT + HT | All received RT, CT and HT. | 1 (0.36%) |
| RT ± S | All received RT, and partial received S. | 1 (0.36%) |
| RT ± CT ± HT ± S | All received RT, and partial received CT, HT and S. | 1 (0.36%) |
| Total |  | 275 (100.00%) |

RT: Radiotherapy; CT: Chemotherapy; HT: Hormone therapy; S: Surgery.

Supplementary Table 3. The treatment types in Expression data

| Treatment type | Meaning | No. of Research (%) |
| --- | --- | --- |
| RT + CT + S | All received RT, CT and S. | 133 (26.60%) |
| RT | All received RT. | 91 (18.20%) |
| RT + CT | All received RT and CT. | 90 (18.00%) |
| RT ± CT | All received RT, and partial received CT. | 66 (13.20%) |
| RT + S | All received RT and S. | 50 (10.00%) |
| RT + CT ± S | All received RT and CT, and partial received S. | 23 (4.60%) |
| RT ± CT ± S | All received RT, and partial received CT and S. | 13 (2.60%) |
| RT ± CT + S | All received RT and S, and partial received CT. | 11 (2.20%) |
| RT ± HT | All received RT, and partial received HT. | 7 (1.40%) |
| RT ± S | All received RT, and partial received S. | 5 (1.00%) |
| RT + HT | All received RT and HT. | 5 (1.00%) |
| RT ± CT ± HT + S | All received RT and S, and partial received CT and HT. | 2 (0.40%) |
| RT + CT ± HT + S | All received RT, CT and S, and partial received HT. | 2 (0.40%) |
| RT ± CT ± HT ± S | All received RT, and partial received CT, HT and S. | 1 (0.20%) |
| RT ± CT ± HT | All received RT, and partial received CT and HT. | 1 (0.20%) |
| Total |  | 500 (100.00%) |

RT: Radiotherapy; CT: Chemotherapy; HT: Hormone therapy; S: Surgery.
