# Supplementary figures and images for: RTPDB: a database providing associations between genetic variation or expression and cancer prognosis with radiotherapy-based treatment
Source: Database (Oxford). 2018 Oct 30;2018:bay118. doi: 10.1093/database/bay118 (PMC6206893; doi:10.1093/database/bay118)

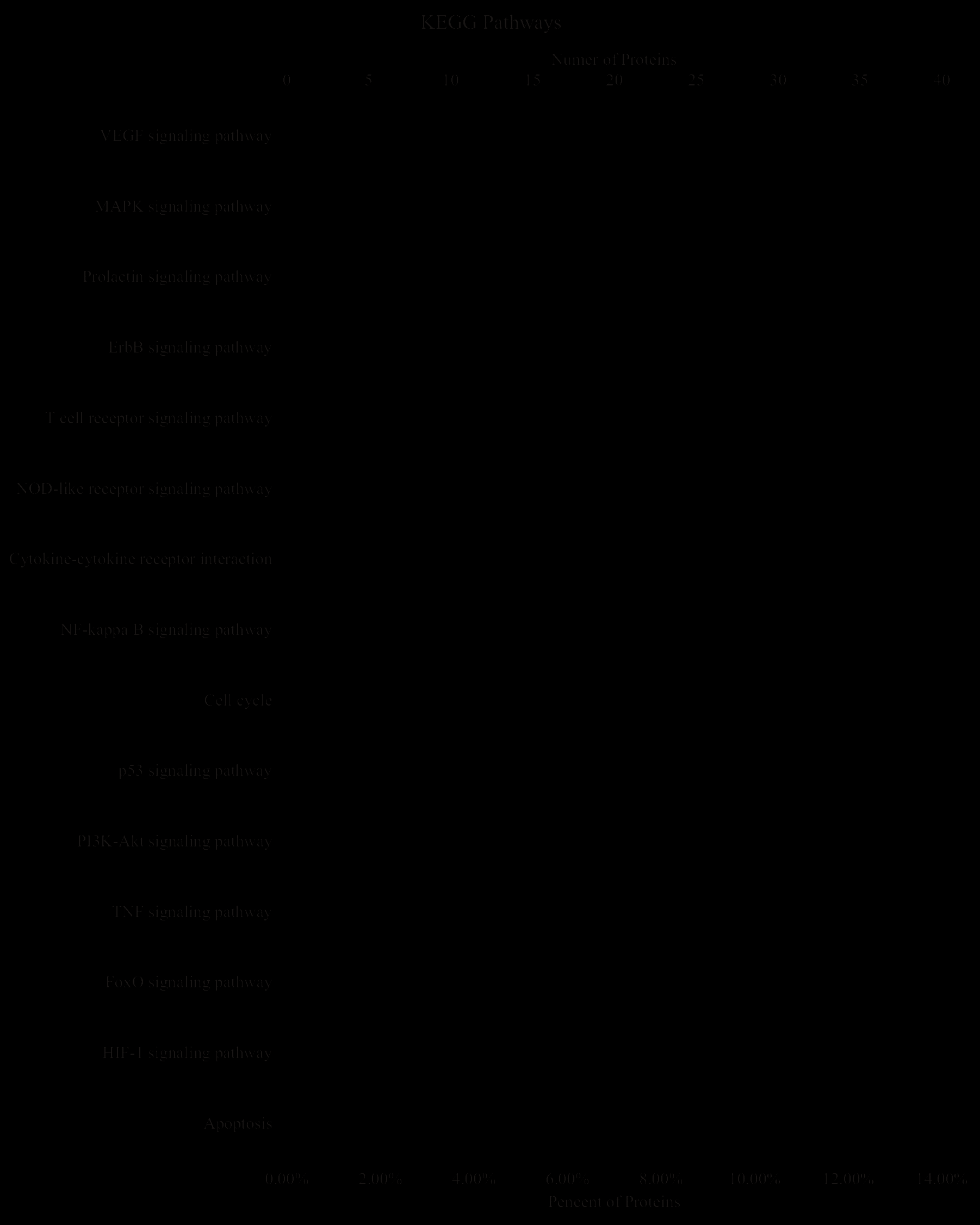

Supplement: Supplementary Data [file bay118_supp.zip › Supplementary Figure 2.tif]

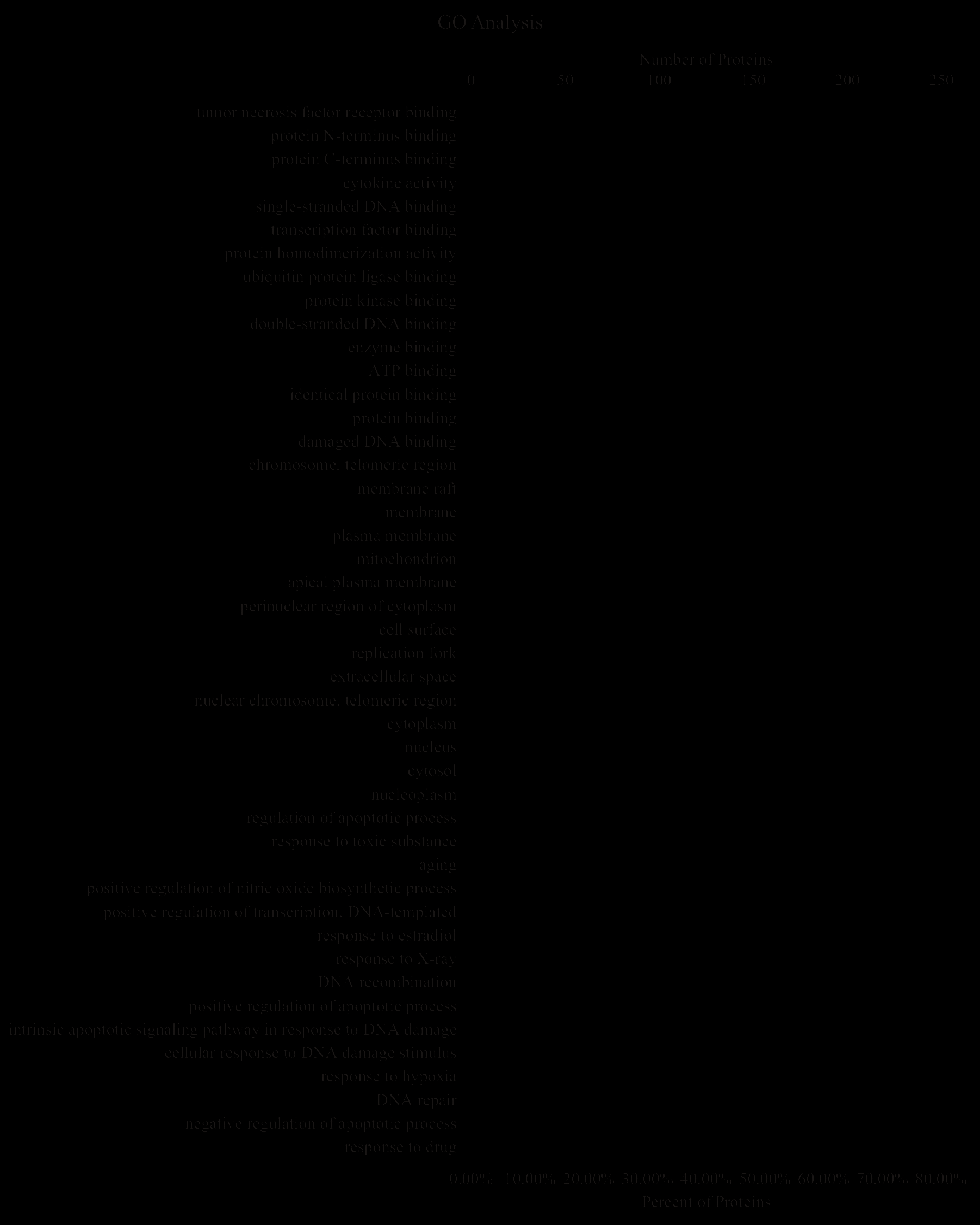

Supplement: Supplementary Data [file bay118_supp.zip › Supplemenrary Figure 1.tif]
